# Health Care Professionals’ Experiences With the Use of Video Consultation: Qualitative Study

**DOI:** 10.2196/27094

**Published:** 2021-07-21

**Authors:** Nina Primholdt Christensen, Karen Emilie Skou, Dorthe Boe Danbjørg

**Affiliations:** 1 Hematological Research Unit Odense University Hospital Odense C Denmark; 2 Department of Hematology Aarhus University Aarhus N Denmark; 3 Centre for Innovative Medical Technology, Department of Hematology Odense University Hospital University of Southern Denmark Odense C Denmark

**Keywords:** video consultation, hematology, outpatient clinic, telehealth, doctor’s perspective

## Abstract

**Background:**

The number of remote video consultations between doctors and patients has increased during the last few years and especially during the COVID-19 pandemic. The health care service is faced with rising rates of chronic illness and many patients who are more confident in self-management of their illnesses. In addition, there is an improved long-term outlook for serious conditions, such as cancer, that might require flexibility in everyday life.

**Objective:**

This study aimed to investigate how medical doctors in the outpatient clinic use and experience the use of video consultations with hematological patients, with a focus on relational and organizational aspects.

**Methods:**

The study was designed as an explorative and qualitative study. Data were collected via participant observations and focus group interviews with medical doctors.

**Results:**

The study identified possibilities and barriers in relation to adapting to the alternative way of meeting patients in the clinical setting. One of the main findings in this study is that the medical doctors were afraid that they missed important observations, as they were not able to perform a physical examination, if needed. They also emphasized that handshake and eye contact were important in order to get an overall impression of the patient’s situation. It also became clear that the medical doctors used body language a lot more during video consultation compared with consultation in a physical setting. The medical doctors found the contact with the patients via the screen to be good, and the fact that the technology was working well made them feel comfortable with the video consultation.

**Conclusions:**

In this study, we found that the medical doctors were able to maintain good contact with the patients despite the screen and were able to assess the patients in a satisfying manner. However, there were still uncertainties among some doctors about the fact that they could not examine the patients physically. New knowledge about how to use gestures and body language during video consultation was obtained.

## Introduction

### Background

The number of remote video consultations between doctors and patients has increased during the last couple of years and especially during the COVID-19 pandemic [1,2]. Telemedicine is an important tool in health care when physical contact needs to be avoided. Politicians see technology-supported health care, such as the use of apps and video consultations, as a solution to the complex problems of demographic development. Delivering health care to an aging and diverse population is a challenge due to the increase of the aging population and the diminishing number of health care professionals [3].

The health care service is faced by not only rising rates of chronic illness, but also many patients who are more confident in self-management of their illness. In addition, there is an improved long-term outlook for serious conditions, such as cancer, that might require flexibility in everyday life [4]. At the same time, there has been focus on centralizing the health care system, in order to attain the most efficient and specialized hospitals in Denmark [5]. This development means, among other things, that patients often must travel further to get to the hospital. Hematological patients in Denmark may spend several hours traveling to the hospital for a consultation [6]. It is therefore relevant to implement telemedicine solutions for patients to spend less time travelling back and forth to visit the hospital. Patients welcome telemedicine initiatives, and a Danish study identified that patients valued the freedom they got when being able to keep up a normal everyday life, take responsibility for their own course of treatment, and feel active, despite their illness [7]. These findings reflect the overall research within telemedicine, where the use of telemedicine has shown positive aspects [4,7-10].

The pandemic has resulted in the natural use of telemedicine [11]. Telemedicine has the potential to deliver health care at a distance, with the potential to improve access to health care and change the way health care is organized. However, the implementation and use of telemedicine can often lead to resistance among health care professionals, as they see it as a threat to their clinical work and professionalism. Many health care professionals still prefer face-to-face communication when interacting with patients and their relatives. A systematic review showed that the difficulties in implementation are linked to the culture among health care professionals. Resistance to change in working procedures and unwillingness to invest time in training for new workflows were significant barriers [12,13]. According to Ross et al, possible difficulties should be identified early in the process when planning implementation of new routines. This will allow preparation of initiatives to prevent resistance to change [14]. Another systematic literature review also identified barriers to the use of telemedicine. The study showed that technology-challenged staff and resistance to change were the most frequent barriers [12].

In the future, the intention of hospitals will be to have fewer admitted patients and instead treat patients in their own homes [15]. In the Region of Southern Denmark, the goal is to convert 30% of outpatient consultations to digital consultations [16]. As described in the above articles, the need for the use of telemedicine is expected to increase in the coming years. It is important to gain knowledge of the use of video consultation from not only the patient’s perspective, but also the medical doctor’s perspective, and in particular, how the use of video consultation can benefit patients.

### Aim

The overall aim was to explore how medical doctors use and experience the use of video consultations. This was done using the following research questions: (1) How do medical doctors use video consultation? (2) How do medical doctors experience the use of video consultation instead of face-to-face consultation? (3) How do medical doctors experience the technical aspects of video consultation? (4) How do medical doctors experience the roles and relation to patients during video consultation?

## Methods

### Design

The study was designed as a qualitative and explorative intervention study. Data were collected using participant observations and focus group interviews.

### Setting

This project was part of a larger pilot study, where patients with hematological diseases from a small island to the south of Funen were given the opportunity for video consultation. The patients could choose to have their consultation with a hematologist from the outpatient clinic in Odense through a video screen, instead of a face-to-face consultation. The patients would be seated in front of a video screen placed at the local hospital on the island, where site staff could help them connect with the doctor via the screen. The pilot study was introduced in April 2017 and continued until the end of December 2017. A total of 17 patients with different hematological diagnoses were included in the pilot project, which made the sample of informants very varied. 

The intervention was initiated as a collaboration between the municipality on the island, the Innovation Department at Odense University Hospital, and the Hematological Research Unit at Odense University Hospital. Two identical video screens were purchased for use at the hospital on the island, and one screen was placed in the outpatient clinic in Odense.

The video consultations had two purposes, namely, monitoring and treating the patients. The medical doctor would use the video consultation in combination with blood results to determine whether the patients were eligible for the next treatment or needed to receive a different treatment for the diagnosis.

The patients were informed by a research nurse from the Hematological Research Unit about participation in video consultation. The research nurse would assist when the video consultation was initiated with the hematology specialist. At the local hospital on the island, the patients could choose to have a nurse participate during the consultation, if they articulated a need for this.

### Sample

The study population consisted of medical doctors who participated in video consultations with patients with hematological diseases, living on a small island south of Funen. The medical doctors who participated in the focus group interview all worked at the Hematological Department at Odense University Hospital and had between one and five video consultations with the hematological patients. The medical doctors did not receive any introduction or guidelines regarding how to behave in front of the patients before, during, and after the video consultations. The medical doctors’ experiences are grounded in their own first-hand experiences.

### Inclusion Criterion

The inclusion criterion for participating in the focus group interview was participation in at least one video consultation with hematological patients form the island to the south of Funen.

### Data Collection

#### Participant Observations

Participant observations of the medical doctors during video consultations were conducted from November 2017 to December 2017. We conducted 5 hours of participant observation.

The last author observed the medical doctors. Green and Thorogood described how observational studies allow us to obtain knowledge about what the observed participants say and what they do in relation to the specific situation [17]. Here, the participant observations allowed us to obtain knowledge about what the medical doctors said and did during the video consultations with the patients [17].

The observations were inspired by James Spradley’s description of passive participation and moderate participation [18]. The observations followed an observation guide that was designed using Spradley theory for observations (Textbox 1).

Passive participation was used in relation to the observations made during the pilot study period, when the video consultations were tested and implemented. The last author listened to and observed the medical doctors’ work with the video consultation and the settings around the video consultation. The observations provided an opportunity for informal interviews with the medical doctors, immediately after the video consultation. Here, it was possible to talk to the medical doctors about their overall impression of the consultation, which provided us with their first-hand impressions [19].

**Participant observation and video consultation from the physician’s end.**

**The physician and the encounter with the technology**

How does the physician handle the technical side of things with the video screen?

Does it seem easy or cumbersome for the physician?

What does the physician comment on/say about the screen/technology?

Does the physician prepare beforehand (fixes hair or clothes, checks phone, etc)?

Are there any technical problems?

How is the sound? How is the image quality?

**Physical setting**

How is the physician placed in relation to the screen?

What does the room look like?

What does the physical setting look like? Is the room hot/cold?

What is the light like?

**Conduct before, during, and after the video consultation**

How does the physician act, verbally and nonverbally?

Does the physician act naturally during the video consultation?

Is there eye contact during the entire consultation?

#### Focus Group Interview and Interview Guides

In January 2018, the first and last authors conducted a focus group interview with seven doctors (two male and five female doctors) with different experiential backgrounds. The focus group interview lasted approximately 90 minutes and took place in a conference room at Odense University Hospital. The last author facilitated the focus group session. The first author was present as an observer, wrote down field notes from the session, and validated the content of the session.

The focus group interview was chosen, as focus group discussions can mobilize associations,* *where the group dynamic contributes to the creation of narratives [20]. It allowed the researchers to get knowledge from the interactions between the medical doctors with different academic experiences. The authors found it relevant to create a focus group of medical doctors that was not too homogeneous and to facilitate discussions between the medical doctors [18].

Before the focus group interview, an interview guide was created [19,21], based on themes from the participant observations and the semistructured interviews with the patients from an earlier Danish study (Multimedia Appendix 1) [7]. The authors also used photo elicitation as a data collection technique. Photos were taken during the participant observations, capturing the video consultations with the patients. They were used as an activity to make the medical doctors reflect on their experiences with the video consultations during the focus group interview [22].

The medical doctors were, at the beginning of the session, asked to write down three positive and three negative thoughts about their experiences with the use of video consultation. Afterwards, they were asked to discuss their experiences with each other. Furthermore, quotes from the semistructured interviews were read aloud to make the medical doctors reflect on and discuss the patient statements.

### Analysis

This study draws on a qualitative research tradition, which is adequate when seeking knowledge about subjective practice experiences, individual patient and professional perspectives, and interactional processes and dynamics involving technology as an actor [23]. The study draws on Don Ihde’s hermeneutic-postphenomenological framework, making it possible to gain an understanding of the interaction between technology, humans, and their lifeworld. Ihde’s methodology will be used to explore how the technological mediation of human practice shapes user experiences and how users attach meaning to certain experiences [24,25]. The analysis process was organized by following the steps from “systematic text condensation” [26-29]. The analysis was organized according to the steps in the systematic text condensation, as shown in Multimedia Appendix 2.

First, we gained an overall impression of the data. This gave us a preliminary set of main themes. Second, the data were broken into meaningful topics, relevant for the research question. Next, the meaningful topics were coded. The first and second authors coded individually, and then, they discussed each topic and the coding, and reached an agreement. Thereafter, the topics were condensed.

As the last step of the analysis, we synthesized the data by applying a shift from condensation to categories. We developed the codes based on the initial themes that were identified in the first step and the theories we applied.

To increase the validation, the analysis was prepared as a cooperation between the first and second authors. Their overall impressions of the data were discussed thoroughly, meaningful topics were highlighted, and codes were applied. The analysis was written by the first author. Then, all three authors met and discussed the findings in relation to relevant literature and the postphenomenological framework, while focusing on technology-mediated transformation, constitution, and perception.

### Ethics

During data collection, the researchers were continuously reflective about the aim of the study and the methods applied. Furthermore, the participants were informed thoroughly about the project, allowing them to make an informed choice as to whether to participate in the study. The participants were informed both orally and in writing about the study and were included after providing their informed consent in compliance with the Helsinki Declaration [30,31]. The participants were guaranteed anonymity throughout the process. The study was registered with the Danish Data Protection Agency (2012-58-0018), and the data were stored at a secure SharePoint site.

## Results

### Overview

During the pilot study, 41 video consultations were performed with patients aged 55 to 85 years with different hematological diagnoses. All patients were in a stable period of their disease during the time when the video consultations took place.

In total, 12 doctors participated in this study. Seven medical doctors, who all had completed between one and five video consultations with different patients having different diagnoses, attended the focus group interview. Five medical doctors were observed during video consultations at Odense University Hospital and participated in informal interviews (Table 1).

**Table 1:** Baseline characteristics of the doctors who participated in the focus group interview.

Doctor ID	Age (years)	Sex	Seniority
Doc 1	43	Female	Doctor in a main education position
Doc 2	41	Female	Doctor in a main education position
Doc 3	31	Male	Doctor in a main education position
Doc 4	52	Female	Chief physician
Doc 5	38	Female	Doctor in a main education position
Doc 6	60	Female	Managing chief physician
Doc 7	45	Male	Chief physician

Results from the focus group interview and the participant observations revealed themes reflecting the medical doctors’ use of and experiences with video consultations.

The themes were “Connecting at a distance – words, body language, laughter, and eye contact,” “Can’t touch this,” “A handshake is not just a handshake,” and “Adjusting to the transformation.”

The themes have been presented and described in more detail below.

### Connecting at a Distance – Words, Body Language, Laughter, and Eye Contact

The analysis of the focus group interview and the participant observations revealed that the medical doctors found it easy to assess and sense the patient’s current condition, despite the consultation being digital. Most of them agreed that verbal connection with the patients was important when seeing the patients.

Comparison of the field notes revealed that both the medical doctors and patients appeared relaxed. The analysis of the field notes showed that the medical doctors were acting confidently and were comfortable with sitting in front of the video screen, as they had good and relaxed interaction with the patients through the screen, characterized by laughter and small talk with the patients. One field note was as follows:

Laughter. Chatting about the weather. Medical doctor sits in front of the screen, relaxed body language. Looking at the screen, smiling.

The statements of the medical doctors who identified that they got a good sense of the patients supported the observations. 

So, I almost think that the technology is so good that you actually sense them quite well. [Female doctor, 43 years old] 

We also found in the data material that a few of the medical doctors mentioned that they found it difficult to maintain eye contact with the patients through the screen. The analysis showed that the medical doctors experienced that they could not achieve the same eye contact as when they were sitting next to the patients in the consultation room. 

No, because it is not there. You can never make eye contact with someone inside a screen. [Female doctor, 52 years old] 

Yet, the analysis also indicated that some doctors experienced that they had eye contact with the patients, and some questioned the need for “physical” eye contact.

I think that sounds right enough, but I do not think it’s such a big problem.

Male doctor, 38 years old

Overall, we can derive that the medical doctors experienced the contact with the patients during the video consultation as more relaxed, because the patients were not stressed, as they remained on the island close to home and did not have to worry about time and transportation.

I also sometimes think that you can actually have better contact via the screen, because I think if you are a little behind and the patients are pressured, because they have to catch the car and the ferry and all that. Then you sometimes have extremely bad contact, because we have only 7.5 minutes left, and sometimes it might be a quarter of an hour that was necessary. Yes, dialogue and contact via the screen is good, because then there are not all these stressful moments.

Female doctor, 41 years old

The analysis of the observations revealed that the medical doctors were using their body language a lot. They waved to say hello and goodbye to the patients, and the patients did the same. They also used their arms and fingers to illustrate what they said, looked straight into the screen while talking, and were laughing with the patients. One field note was as follows:

When the patient talks, the doctor leans towards the screen, focusing on the screen. Looking directly at the screen, slightly furrowed eyebrows. The patient also leans all the way to the screen, looking at the doctor.

November 16, 2018; 11 am

The data from the focus group interview also supported these observations.

I have not met my patient in real life. I have only met her on the screen, and we have done so 3 times. … So, we're starting to know each other and waving goodbye and stuff like that… yes.

Female doctor, 43 years old

Yet, the analysis also indicated that for some of the medical doctors, it was important to know and see the patients before the first video consultation. Therefore, it seems to be a subjective perception and not related to the technology.

Yes, it requires that it is the same doctor who has met them physically before, I would say, so you kind of know who it is.

Female doctor, 38 years old

### Can’t Touch This

For medical doctors who are used to using their hands when examining patients, it is a big change not to be able to use the sense of touch. It transforms the consultation because they cannot use “a tool” that they normally use when seeing patients.

In general, throughout the analysis, it became clear that it was challenging for the medical doctors to get used to the new practice with video consultation instead of physical consultation, where the doctors would normally be able to examine the patient physically, if required. The doctors felt that they missed some important information when they were unable to observe and examine the patients in the usual way. Some of the medical doctors were also afraid that they might miss how the patients’ general condition develops.

We risk exactly those 80-year-old patients with myeloma in the stable phase. We will end up following them for 10 years, and they are in a nursing home and they put a screen in front of them, and it makes no sense if we are phoned and say, no the patient cannot do this trip to the hospital. And if we get that phone call, then we can say well, then we do not have to take blood samples, and if the patient doesn´t manage the trip, then you never get in chemo, but she manages well to get a screen in front of the her. The monitoring will be dragged out for too long.

Male doctor, 42 years old

The analysis identified that the medical doctors observed and examined the patients in many different ways and that the medical doctors are not always aware of what tools they use to diagnose and evaluate the patients. They do several things instinctively.

Yes, here you become aware that there are some things we do subconsciously.

Female doctor, 52 years old

The medical doctors found the possibility of performing a physical examination important in the consultation with the patients. The medical doctors were therefore concerned by the fact that they were unable to perform an examination during the video consultation. However, during the project period, only a few doctors needed to see the patients physically following the video consultation. It became evident that it was a problem if the patients had a new medical condition that they wanted the medical doctors to investigate. One fieldnote was as follows:

The patient tells of itchy rash. The doctor points to her forearm and says that the patient should show her forearm. The doctor leans all the way to the screen. She says, yes it´s hard to see on the screen, and I can´t touch it.

November 16, 2018; 11 am

Some of the medical doctors also expressed that during the video consultation they were able to retrieve important information through the screen, which is opposite to telephone contact with no visual contact. Via the video consultation, the doctors got indirect information, for example, facts about how the patient and the surroundings appear.

I just think the video provides something else than just a voice, that is. Of course, you do not see the patient sitting in the waiting room, but you still get an impression of whether it is someone who is sitting with their hair hanging in a mess, someone who is still sitting in a dressing gown - you get a picture of that via the video.

Female doctor, 56 years old

The analysis indicated that the medical doctors were afraid of missing important details about the patients when they could not be hands on with them, as they could during a physical consultation. However, at the same time, the medical doctors also expressed that they experienced that the patients were very satisfied with the video consultation.

And the patients are satisfied. They really are. And as you also say, there is a good quality of sound and picture. And it goes both ways. The patient is also very focused when we talk.

Female doctor, 52 years old

The quality of the sound and picture during the video consultations was satisfying and contributed to an overall secure feeling for the doctors, because they could see the patients as clearly as if it was a face-to-face consultation.

I think there is a good sound and picture quality. So that way it seems very real.

Female doctor, 43 years old

### A Handshake is Not Just a Handshake

The analysis revealed that a handshake gives the doctors a lot of information about the patients. Many of the medical doctors also expressed that they missed the physical handshake and being able to take in the general appearance of the patients, though the connection with the patients was sufficient.

Then I might also miss a bit… that handshake you have at the consultation, where you get a feeling of what kind of patient who comes in. Is it someone who is nervous or is it someone who is strong? [Female doctor, 43 years old] 

Some of the objective parameters that the doctors used in their overall assessment of the patients were found to be difficult to recreate during the video consultations. It is clear that the way a doctor senses a patient is from the “whole experience.”

But I am also thinking about what you said. You don´t get the handshake and you can’t follow them walking to the waiting room. Those 8 steps to see if they jump out of the chair or… They are sitting in that chair – and there you can look reasonably fresh and cheat. Or not cheat. You will get a more positive view than you would, if you saw them in real life, when they walk to the consultation room? You lose some objective information about the patient when you only see them via the screen. We see so many different patients, there is no continuity, so I would miss seeing them physically.

Female doctor, 38 years old

The analysis points to important aspects. The handshake and the way patients walk into the room are important observations for doctors. Even though the consultation is mostly “talk,” the movements and handshake reveal important information, which is necessary to address when discussing telemedicine. The fact that video consultation is not a 3D experience is a big change, and therefore, doctors need to redefine and reinvent the way they see patients.

But it's probably also something to do with the fact that we have been used to the consultation starting when you meet the patient out there, and you observe how the handshake is, how they get up from a chair, how fast their disease develops. So, it feels completely wrong that this part is missing.

Female doctor, 43 years old

### Adjusting to the Transformation

Seeing patients through a screen changed something for the doctors. They all experienced it as a challenge, and they emphasized that they need help with adapting to the new types of consultations. The medical doctors experienced that they somehow were making their own guidelines for video consultation, as they needed guidance and they had not been introduced to guidelines on how to conduct a video consultation.

The medical doctors considered it relevant to receive instructions on how to navigate in front of the screen before the initiation of video consultations in the outpatient clinic.

I´ll be surprised if someone hasn´t found tips and tricks to say “hello” and “goodbye” in a decent way. You throw yourself into it, like everything else. But for sure, some might say that there might be some standard practices or something.

Female doctor, 41 years old

The medical doctors experienced situations where they acted differently in the video consultation when compared with a traditional face-to-face consultation. One example was when another person, besides the patient, was visible during the video consultation.

It was not expected that someone would sit next to the patient during the consultation. It was exactly the point, that there shouldn´t?

Female doctor, 52 years old

Sometimes there is a person sitting who has not participated as such in the conversation, but where I have just thought; who are you?

Female doctor, 41 years old

The physical framework was important in how the doctors experienced the consultation and how they placed themselves in front of the screen.

There is also something about how you place yourself in the room. Maybe we could try that some more.

Female doctor, 43 years old

The doctor is adjusting the camera. The doctor sits straight in front of the screen. Obviously aware to sit in an angle, so the patient can see her. The doctor smiles and uses facial expressions. Emphasizes the words, talks a bit slowly, hesitant with a nod, sees if the patient wants to say something before, she continues talking.

Field note; November 24, 2018; 12:45 pm

In continuation of the physical framework, the video consultations also involved some technical aspects regarding how to show the patient the laboratory results and how the doctor should be placed in the room, so the patient can see the doctor and the surroundings.

And sometimes I like the fact when I have the patient in front of me, to turn the screen and show the blood results. And you don´t do that the same way via the video consultation. But it is probably also something that can be solved technically.

Female doctor, 43 years old

## Discussion

### Principal Findings

This study explored how medical doctors used and experienced the use of video consultation in the outpatient clinic instead of or as a supplement to face-to-face consultation. The study identified possibilities and barriers in relation to adapting to the alternative way of meeting patients in the clinical setting. One of the main findings in this study is that the medical doctors were afraid that they missed important observations, as they were not able to perform a physical examination, if needed. They also emphasized that a handshake and eye contact were important to get an overall impression of the patient’s situation. It also became clear that the medical doctors were using body language a lot more during video consultation compared with physical consultation. The medical doctors found the contact with the patients via the screen to be good, and the fact that the technology was working well made them feel secure with the video consultation.

### Video-Mediated Contact

In many Western cultures, the most common way to initialize a social interaction is a handshake between people [32]. In Denmark, the handshake has several meanings and holds a long tradition. The handshake is often debated, as in some other cultures, it is not socially acceptable for a man and a woman to shake hands. Yet, the handshake is essential in the Danish culture and is also a subject of political debate. Shaking hands with the patients was not possible during the video consultations. The question about the handshake has a new perspective owing to the current COVID-19 pandemic. It is no longer possible to shake hands with your doctor, and it will be interesting to observe how this will affect the doctor-patient relationship in the long run [2].

We also found that the medical doctors were using body language a lot during the video consultations. They waved to the patients to compensate for the missing handshake. We know from another study of video consultations that patients also wave or use facial expressions when talking to their doctors [7]. The study revealed that it was not an issue for the patients as they would compensate the missing handshake with a wave or with enhanced facial expressions. The study showed that having the contact mediated through a screen invited the participants to use other gestures. Ihde explained that technology is shaping our experiences of a situation and invites humans to act in certain ways [24]. In the specific situations with video consultations, the technology invited the participants to greet each other in a different way than when meeting face to face. This compensated for the lack of physical contact.

A well-known fear of technology is that machines will replace human contact, making care “cold” by reducing it to mechanical interactions with machines [31]. Yet, the results of a previous study showed that the patients experienced intimacy at a distance, even though they could not touch the doctor with a physical handshake [7].

In the actual study, the technology invited the medical doctors to greet the patients in a different way than that when meeting the patients face to face, to compensate for the missing physical presence, and the medical doctors adapted to the new technology. Yet, we found that it was a concern that the medical doctors could not shake hands with the patients, as they, as professionals, used the handshake for medical observations in the form of observations of how the patients walk, smell, and interact. Therefore, it was not only the social interaction that was at stake, but also the medical observation that they would miss when not greeting the patients physically.

Other findings revealed that the medical doctors found video consultation less stressful than traditional consultation at the outpatient clinic, because the patient was not in a hurry to catch public transport, and the overall experience of the meeting with the patient was more relaxed. This finding is supported by other Danish studies, where patients expressed how important it was to not spend time on travelling to the hospital and to have the ability to keep up a normal everyday life [7,13,33,34]. It is very important for patients to keep up a normal everyday life when sick [7].

### Present Without Being Present

In this study, we found that the medical doctors had a professional relation with the patients even though the consultation was through a screen. The medical doctors also experienced better contact with the patients even though the patients were not in the room. In another study by Van Gurp et al, it was noted how teleconsultants managed an empathic patient-professional relation with outpatients receiving palliative care [35].

Van Gurp et al found that the team members chose not to discuss sensitive and emotional topics with vulnerable patients [35]. This choice was made because of the inability to physically comfort the patients, because of the physical distance. However, the same patients reported that a nonphysical professional listener provided the exact freedom they needed to define their own role and co‐design their own care in equal patient-professional relationships [35]. The same feeling of freedom without being physically present was found in a Danish study [7], where the patients felt the same level of intimacy with the medical doctors, even though the contact between them was mediated through a screen. In a Danish study on palliative care, it was found that both patients and relatives were more actively involved in treatment and care when consultations with nurses and medical doctors took place via a screen [33]. In a Swedish study from 2020, it was found that most doctors had a positive attitude toward the use of video consultations and that the reliability of the technical platform was very important for doctors to feel comfortable during the consultation [36].

### The Transition

This study discovered that the technology was easy to use, and the quality of the sound and picture was impressive. However, the fact that a screen was present between the medical doctors and patients posed some challenges as to how the medical doctors would assess the patients. The change from the possibility of examining the patients when required to the fact that it was not an option was challenging for some of the medical doctors. Ihde stated that handling new technology is a learning process and that it is experienced as stressful until the user has learned how to use it for specific practices [26]. Ihde used the term “embodiment” to describe the process that takes place when a certain technology becomes integrated as a useful tool for the ones using it. In another study, where patient rounds with video-consulted relatives was examined, it was found that health care professionals experienced changes regarding workload, culture, and organization when the new way of doing the rounds with the relatives was introduced [13].

The medical doctors missed seeing the patients walk from the waiting room and being able to evaluate their general status. The medical doctors in this study did not receive any instruction as to how to act in front of the video screen or how to use the technology to get the desired and necessary information from the patients. According to Kotter, it is important to give a sense of meaning to the individual when facing changes in an organization. Moreover, that the change is experienced as being urgent is important as to how successful the change or implementation will be [37]. In this study, the test of the video consultations was not urgent, and the observations of the medical doctors showed that they tried to navigate in this new setting, but without any guidance. The medical doctors’ motivation for working with the change involving replacing face-to-face consultation with video consultation was the patients’ statements about their satisfaction with the flexibility and the good contact they had with the doctors through the screen [7].

### Strengths and Limitations

Our study was limited by the reduced number of medical doctors participating in the focus group interview, making this a small-scale study. In qualitative research, most studies are typically small scale [17].

The aim of this study was to perform an in-depth exploration of the phenomenon under investigation. Therefore, the intention of this study was to understand and explain medical doctors’ experiences of video consultations with hematological patients located in front of a screen at a small island.

We have provided detailed descriptions of the context of the study, as well as the observations and the doctors’ experiences with video consultations. The results do not show statistical generalizability, but analytical generalization, which emerges by means of the dialectic between theory and practice.

The first two authors conducted the analysis jointly to increase reliability. We presented the analysis process in a table to ensure transparency. Quotations from the data were used to link to the participants’ original statements to ensure validity.

### Conclusions and Implications for Practice

When data for this study were collected, there was a lack of evidence on how medical doctors experience video consultations. At present, after the start of the COVID-19 pandemic, more scientific literature about doctors’ experiences is available. In this study, we found that doctors were able to maintain good contact with their patients despite the screen and were able to assess the patients in a satisfying manner. However, there were still insecurities among some doctors about the fact that they could not examine the patients physically. New knowledge about how to use gestures and nonverbal body language during a video consultation was also discovered.

The new knowledge about how medical doctors can use video consultations will benefit both patients and health care professionals in allowing the health care system to provide more tailored treatment, which will mean improved flexibility for the patients. Understanding how medical doctors experienced the use of the new consultation form can inspire other medical doctors to implement and use video consultations in outpatient clinics or other clinical settings. Especially during the COVID-19 pandemic, it has become imperative to implement video consultations quickly at hospitals, and scientific knowledge sharing has become more relevant than ever. It will also be possible to create guidelines for medical doctors using video consultation as a supplement to normal consultation in the outpatient clinic, with the results of this study as a starting point. Even though the data of this study were obtained before the COVID-19 pandemic, the results can be used as an important supplement for the history of novel experiences with video consultations before the pandemic and can be used for the development of video consultations in the perspective of today’s situation.

## Appendix

Multimedia Appendix 1 Focus group interview guide.

Multimedia Appendix 2 The analysis process, with examples from the analysis.
