# Supplementary material for: Health Care Professionals’ Experiences With the Use of Video Consultation: Qualitative Study
Source: JMIR Form Res. 2021 Jul 21;5(7):e27094. doi: 10.2196/27094 (PMC8339982; doi:10.2196/27094)
Supplement: Multimedia Appendix 1 [file formative_v5i7e27094_app1.pdf]

Multimedia appendix 1. Focus group interview guide.

Introduction

Welcome. I'm happy to see you all, and I look forward to spending the next two hours with you.

My name is Dorthe, and it will primarily be me who directs the interview. I am a nurse researcher, and as you can see, Nina is also present. She will observe and take notes. She will also ask questions, if she becomes curious about what is being said.

Please have a cup of coffee or tea. Make yourselves comfortable.

Is it OK if we take some pictures during the interview? The images may be used for presentation or report in connection with the project.

The interview will take about an hour and a half. It will be recorded by a dictaphone to help us remember what is being said. And I want to emphasize that you will remain anonymous in the project. Everything that's being talked about today will be treated with respect and confidentiality.

We will start with a short presentation, where you can briefly state how many times you have tried video consultation. Then we would like to hear about your experiences and reflections.

We will divide the interview into two sections, where we would like hear your immediate thoughts first, and then you will be discussing video consultation based on some pictures and quotes.

I would like us to start with a small presentation round where you can state your name and your age and possibly job.

Focus group

You've come in for this focus group interview, which is a bit different from what you normally associate with an interview. You will be doing most of the talking, and I would like you to talk among yourselves about some topics that I will be introducing. You will be talking and discussing with each other. You run the discussion yourselves, and you must ensure that everyone gets the opportunity to speak. If the discussion goes off track, or you have nothing to say, someone from the group usually does something about it. Otherwise, I'll help you move along.

There are no right or wrong answers. All experiences are equally important.

Do you have any questions before we get started?

Experience with video consultation (30 minutes)

To get started, you first need to think back to the video consultations you've had.

Please write down three good things about video consultations.

Please write 3 things you miss.

Based on your cues, we will discuss your thoughts on the use of video consultation.

Who wants to start?

Help questions

What was it like to handle the technology? Easy/difficult?

What was the time consumption compared to conventional consultations?

Would you have preferred a “regular” consultation?

What do you think about?

- Eye contact with the patients – patients say they experience more presence?
- Preparation for video consultation/regular consultation?
- Verbal preparation of the patient before the first video consultation?
- Training physicians in the use of video consultation – would that make a difference?
- Lack of physical contact – has it been missed?

Cues: What matters to you when meeting the patient?

20 minutes:

Here, the medical doctors are presented with prompts, which they must relate to.

We print statements/pictures of contact between prof and patient on some cards and place them on the table, so they can be used for starting a discussion.

You must now select some cards with quotes or a picture and state your immediate thoughts regarding the image. Please feel free to take more images/statements as we go.

The idea is that the physicians themselves manage the discussion, which is prompted by the statements and images.

Images

Picture of a physician, bedside, in conversation with a patient.

Picture of a physician sitting far from the patient, looking down at the medical records.

Picture of a monitor.

Picture of two persons in conversation, one with their hand on the other's shoulder.

Picture of someone gesturing.

Picture of a video consultation with several people.

Quotes

Patient quotes from interviews

Presence

Why do I have to show up for controls?

Collaborating with your GP

Do you have any idea of how many resources I spend psychologically on going to OUH? All the sick people I have to deal with.

More infections
