# Supplementary material for: Health Care Professionals’ Experiences With the Use of Video Consultation: Qualitative Study
Source: JMIR Form Res. 2021 Jul 21;5(7):e27094. doi: 10.2196/27094 (PMC8339982; doi:10.2196/27094)
Supplement: Multimedia Appendix 2 [file formative_v5i7e27094_app2.pdf]

Multimedia appendix 2. The analysis process, with examples from the analysis.

**Step 1**<br>Superior themes extracted after the first open reading.	**Step 2**<br>From themes to codes. Identifying meaningful units. The meaningful units are coded based on the superior themes.		**Step 3**<br>From codes to meaning. The meaningful units are sorted into groups.
	Quote	Code	
Habits	They are much more relaxed and yes. But there are some things missing, and I also just think the contact. It's just not the same. And you are most likely just used to the fact that you probably assess them from the moment you get them in the hallway also all the way in – and that is what I miss a little.	Process of change	New roles and opportunities
Eye contact	No, because it is not there. You can never make eye contact with someone inside a screen.	Overall impression	Assessment and sense for the patient
